# Conventional Anti-glioblastoma Chemotherapy Affects Proteoglycan Composition of Brain Extracellular Matrix in Rat Experimental Model *in vivo*

**DOI:** 10.3389/fphar.2018.01104

**Published:** 2018-10-02

**Authors:** Alexandra Y. Tsidulko, Cynthia Bezier, Gabin de La Bourdonnaye, Anastasia V. Suhovskih, Tatiana M. Pankova, Galina M. Kazanskaya, Svetlana V. Aidagulova, Elvira V. Grigorieva

**Affiliations:** ^1^Institute of Molecular Biology and Biophysics, Novosibirsk, Russia; ^2^Novosibirsk State University, Novosibirsk, Russia; ^3^UPMC-Sorbonne Universities, Paris, France; ^4^Institut National des Sciences Appliquées de Toulouse, Toulouse, France; ^5^Meshalkin Novosibirsk State Research Institute of Circulation Pathology, Novosibirsk, Russia; ^6^Novosibirsk State Medical University, Novosibirsk, Russia

**Keywords:** glioblastoma multiforme, temozolomide, dexamethasone, extracellular matrix, proteoglycan, glycosaminoglycan, heparan sulphate, chondroitin sulphate

## Abstract

Temozolomide (TMZ) is a conventional chemotherapy drug for adjuvant treatment of glioblastoma multiforme (GBM), often accompanied by dexamethasone (DXM) to prevent brain oedema and alleviate clinical side effects. Here, we aimed to investigate an ability of the drugs to affect normal brain tissue in terms of proteoglycan (PG) composition/content in experimental rat model *in vivo*. Age- and brain zone-specific transcriptional patterns of PGs were demonstrated for 8, 60, and 120 days old rats, and syndecan-1, glypican-1, decorin, biglycan, and lumican were identified as the most expressed PGs. DXM treatment affected both PG core proteins expression (mainly syndecan-1, glypican-1, decorin, biglycan, lumican, versican, brevican, and NG2) and heparan sulphate (HS)/chondroitin sulphate (CS) content in organotypic brain slice culture *ex vivo* and experimental animals *in vivo* in a dose-dependent manner. TMZ treatment did not result in the significant changes in PG core proteins expression both in normal rat brain hippocampus and cortex *in vivo* (although generics did), but demonstrated significant effects onto polysaccharide HS/CS content in the brain tissue. The effects were age- and brain zone-specific and similar with the age-related PGs expression changes in rat brain. Combination of TMZ with DXM resulted in the most profound deterioration in PGs composition and content in the brain tissue both at core protein and glycosaminoglycan levels. Taken together, the obtained results demonstrate that conventional anti-glioblastoma therapy affects proteoglycan structure and composition in normal brain tissue, potentially resulting in deterioration of brain extracellular matrix and formation of the favourable tumorigenic niche for the expansion of the residual glioma cells. During the TMZ chemotherapy, dose and regimen of DXM treatment matter, and repetitive low DXM doses seem to be more sparing treatment compared with high DXM dose(s), which should be avoided where possible, especially in combination with TMZ.

## Introduction

Glioblastoma multiforme (GBM) is the most aggressive malignant brain tumour (Thakkar et al., 2014; Nørøxe et al., 2017). Despite of numerous new strategies for targeted treatment of glioblastoma, progress in the field remains insufficient (Nam and de Groot, 2017; Touat et al., 2017). After the surgical resection of the tumour node, the main purpose of all consequent treatment strategies is to eliminate the remaining glioblastoma cells and prevent the disease relapse being a main cause of the patient’s deaths.

However, being concentrated to destroy the glioblastoma cells, one can overlook a danger coming from a significant impairment of the surrounding normal brain tissue during the adjuvant therapy. In fact, the most dangerous trait of glioblastoma is its’ active invasion into the surrounding healthy brain tissue (Paw et al., 2015; Diksin et al., 2017), and the invasiveness of GBM cells and tumour development depend on not only migration capabilities of the proliferating glioblastoma cells but also structure of the surrounding normal brain tissue (Song and Dityatev, 2017; Manini et al., 2018). One of the key invasion-related component of normal brain tissue is extracellular matrix (ECM) which occupies near 20% of its volume and serve as a main basic element of tissue structure and physiology (Nicholson and Hrabětová, 2017). ECM is not only a physical non-specific barrier but is actively involved in cell–cell and cell–matrix interactions and signalling through the numerous ligands like chemokines, growth factors, and adhesion molecules (Miyata and Kitagawa, 2017). Unlike in other organs, brain ECM composes mainly of glycosylated molecules such as proteoglycans (PGs) and glycosaminoglycans (GAGs), predominantly chondroitin sulphate PGs (CSPGs) (Silver et al., 2013; Dyck and Karimi-Abdolrezaee, 2015), hyaluronic acid (HA) (Park et al., 2008; Miyata and Kitagawa, 2017), and heparan sulphate PGs (HSPGs) (Yu et al., 2017).

At present, most of GBM tumours are treated according to a common scheme based on maximally safe surgery, followed by a combination of radiotherapy and chemotherapy with temozolomide (TMZ) and dexamethasone (DXM) as accompanying anti-oedema drug (Seystahl et al., 2016; Reitman et al., 2018). During the adjuvant anti-glioblastoma therapy, both the GBM cancer cells and a surrounding normal brain ECM are exposed to those drugs but their effects towards the brain ECM remain unclear.

TMZ, by definition, is a drug to eliminate cancer cells and its’ molecular effects on glioblastoma cells and clinical effects on GBM tumour development are well known (Bei et al., 2010; Yang et al., 2014). However, TMZ effects towards the normal brain tissue surrounding the GBM tumour and its’ ECM (especially PGs) remain completely uninvestigated.

More information can be found in the literature on corticosteroid drug DXM, routinely used to prevent/treat peritumoural brain oedema during GBM chemotherapy, despite significant systemic side effects (Murayi and Chittiboina, 2016). DXM do affects glioblastoma cell biology *in vitro* and tumour development *in vivo*, and an overall impression denotes rather negative action of the drug for GBM prognosis and patients survival. DXM use correlates with low overall survival and progression-free survival of GBM patients (Shields et al., 2015); retrospective clinical analyses in three independent patient cohorts and mouse experimental data suggest that DXM may decrease the effectiveness of treatment and shorten patients’ survival in glioblastoma, highlighting the importance of identifying alternative agents and substantiating the request for restricted use of corticosteroids in glioblastoma (Pitter et al., 2016). However, as to glioma cells *in vitro*, DXM demonstrates opposite effects as it decreases TMZ-induced apoptosis in human gliobastoma T98G cells (Sur et al., 2005); inhibits glioma cell proliferation in a concentration and species-dependent manner and reduces tumour-induced angiogenesis (Fan et al., 2014), decreases MMP-2 secretion and invasiveness of human U87MG glioma cells (Lin et al., 2008) and suppresses the dispersal of GBM cells through the stimulation of fibronectin secretion and inhibition of the glioma cells motility (Shannon et al., 2015). These data suggest that the negative effect of DXM treatment *in vivo* seem to be related to some unknown molecular mechanisms (possible related to microenvironmental issues) rather then its’ direct action towards glioblastoma cells, however, the issue remain uninvestigated.

In this work, we aim to study effects of TMZ and/or DXM treatments on PGs expression and ECM structure in normal rat brain tissue in organotypic system *ex vivo* and animals experimental model *in vivo*.

## Materials and Methods

### Animals

Wistar rats aged 8, 60, and 120 days were used in the experiment on age-dependency (totally 15 animals, 5 animals/group). All other experiments *in vivo* were performed on male Wistar rats aged 9–10 weeks and weighing 200–250 g at the beginning of the experiment (totally 35 animals). Animals were housed in polycarbonate cages (36 × 50 × 28 cm) with free access to food and water, natural light/dark cycle, temperature of 25 ± 1°C, humidity of 50–60% and weighed once/day. All the studied animals were adapted to the experimenter for 5 days prior to the start of the experiment. All procedures were conducted in accordance with European Communities Council Directive 2010/63/EU. All efforts were made to minimise animal suffering and to reduce the number of animals used. Animals were sacrificed by decapitation using guillotine according AVMA Guidelines for the Euthanasia of Animals (American Veterinary Medical Association, 2013).

### Organotypic Hippocampal Slice Culture

Organotypic hippocampal slice cultures (OHSCs) were prepared according to the previously described protocols (Gähwiler et al., 1997) with modification (Palyanova et al., 2013). Briefly, neonatal Wistar rat pups (post-natal day 7–8) were decapitated, the brains were rapidly removed under aseptic conditions and placed into ice-cold Hank’s balanced solution. The hippocampi were removed and placed into agarose blocks and cut rapidly with a tissue chopper into 400 μm transversal slices. The slices were transferred to collagen-coated coverslips and placed into Petri dishes containing specialised pedestals. Hank’s solution was placed on the bottom of the Petri dishes to supply additional humidity. 100 uL of culture medium, consisting of 25% Hank’s balanced solution, 65% DMEM, and 10% foetal bovine serum, was added to each cover slip. The OHSCs were cultivated in a 90% humidified atmosphere with 5% CO2 at 36°C. The medium was changed twice a week; the state of the OHSCs was controlled visually. At day 7 of incubation, DXM was added to the culture medium to final concentrations of 10 nM–200 μM for 24 h. To determine the effects of DXM on OHSC, the cultures were collected into RNAlater solution and used in RT-PCR analysis. All experimental procedures involving rats were approved by the Institutional Animal Care and Use Committee and performed according to the Directive 2010/63/EU.

### Drug Administration

Rats were randomly divided into groups (5 animals/group). The synthetic glucocorticoid agonist DXM (KRKA) was administered subcutaneously (s.c.). Two groups of rats received a single injection of DXM (0.1 or 5 mg/kg) and were sacrificed after 24 h to determine the short-time effects of the drug. Third group received 1 mg/kg of DXM daily for 1 week. TMZ-based drugs (Temodal, Temozolomide-Teva, and Temozolomide-Rus) were administrated peroral 30 mg/kg per day for 5 days. Control group received saline injections (s.c.) of the same volume as the experimental group. Animals were sacrificed by decapitation and one hemisphere from each animal was collected in RNAlater for further RT-PCR analysis, while the other one was fixed in 4% paraformaldehyde and embedded into paraffin blocks.

### RT-PCR Analysis

Total RNA was extracted from the brain samples using the TRIzol Plus RNA Purification Kit (Thermo Fisher Scientific, United States) according to the manufacturer’s instructions. cDNA was synthesised from 1 μg of total RNA using a First Strand cDNA Synthesis kit (Fermentas, United States) and 1/10th of the product was subjected to PCR analysis. Quantitative RT-PCR (qRT-PCR) was performed using the CFX96^TM^ Real-Time PCR Detection System (Bio-Rad, United States) and the Taq-pol (IMCB, Russia) Maxima SYBR Green/RO master mix (Thermo Fisher Scientific) under the following conditions: 95°C for 3 min, followed by 40 cycles at 95°C for 10 s, 59°C for 20 s, and 72°C for 30 s. The total reaction volume was 25 μl. The relative amount of mRNA was normalised against Gapdh mRNA, and the fold change for each mRNA was calculated by the 2^-ΔCt^ method. Primer sequences for rat proteoglycan genes are presented in **Table 1**.

**Table 1:** Sequences of primers used in PCR analysis.

Gene	Sequence
*Sdc1*	5′-GAACCCACCAGCAGGGATAC-3′
	5′-CACACTTGGAGGCTGATGGT-3′
*Gpc1*	5′-GCCAGATCTACGGGGCTAAG - 3′
	5′-AGACGCAGCTCAGCATACAG-3′
*Hspg2*	5′-TGATGACGAGGACTTGCTGG-3′
	5′-ACACCACACTGACAACCTGG-3′
*Vcan*	5′-ATGTGGATCATCTGGACGGC-3′
	5′-GTTTCGATGGTGGTTGCCTC-3′
*Bcan*	5′-AGGGGACCTCACAAGTTCTTC-3′
	5′-ATTTGACTCGGGGAAAGCCC-3′
*Cspg4*	5′-ATCTGGGAGGGGGCTATTGT-3′
	5′-GTACGCCATCAGAGAGGTCG-3′
*Dcn*	5′-AATGCCATCTCCGAGTGGTG-3′
	5′-TTGTCGTGGAGTCGAAGCTC-3′
*Bgn*	5′-GAACAGTGGCTTTGAACCCG-3’
	5′-CCTCCAACTCGATAGCCTGG-3′
*Lum*	5′-AATTTGACCGAGTCCGTGGG-3′
	5′-GCCTTTCAGAGAAGCCGAGA-3′
*Gapdh*	5′-ATGGCCTTCCGTGTTCCTAC-3′
	5′-TCCAGGGTTTCTTACTCCTTGG-3′

### Immunostaining

For immunohistochemistry, 3,5-μm sections of formalin-fixed paraffin-embedded samples were deparaffinised in xylene twice for 5 min following stepwise rehydration in 100, 95, and 70%, ethanol for 5 min each and 5 min in deionised water. Antigen retrieval was performed at 99°C for 20 min in citrate buffer (pH 6.0). Non-specific binding was blocked with 1% BSA and 10% foetal bovine serum in phosphate buffered saline (PBST) at room temperature for 1 h. After blocking, the slides were incubated with anti-decorin (Abcam ab175404), anti-syndecan-1 (Abcam ab34164), anti-NG2 (Abcam ab83178), anti-HS (Millipore MAB2040), and anti-CS (Sigma-Aldrich C8035) primary antibodies for 1 h at room temperature. After rinsing with PBST three times for 15 min, slides were incubated with secondary antibodies anti-mouse Alexa Fluor 488 (Abcam ab150117) or anti-rabbit Alexa Fluor 647 (Abcam ab 150063) at room temperature for 1 h. After PBST rinse 3 × 10 min, slides were mounted using SlowFade Gold (Thermo Fisher Scientific, Untied States) mounting medium with DAPI and imaged on a Confocal Laser Scanning Biological Microscope Fluoview FV1000 (Olympus, United States). The images were processed using background subtraction to remove shading due to non-uniform illumination and inhomogeneous staining effects and using colour compensation to minimise the effects of spectral bleed-through among the three-color channels (red, green, and blue). Quantitative analysis of the images was performed using CellProfiler 2.2.0 software (Lamprecht et al., 2007).

### Statistical Analysis

Statistical analyses were performed using ORIGIN 8.1 software; a value of *p* < 0.05 was considered to indicate a statistically significant difference. Data are expressed as the means ± SD.

## Results

Conventional anti-glioblastoma chemotherapy includes TMZ as a basic “first-line” drug accompanied by supporting therapy with DXM to prevent brain oedema. In spite of the established effects of the drugs towards cancer cells, their effects to the surrounding normal brain tissue need additional investigation and dictate specific methodological approach. Cultured cross-sections of rat hippocampus *ex vivo* (OHSC) were used instead of GBM cell culture model *in vitro*, as it represents 3D structure of brain tissue and can be used for ECM study (Graber et al., 2012; Minami et al., 2017). In this study, DXM only but not TMZ effects were investigated in the OHSC experimental model *ex vivo* because the last one represents pro-drug and needs to be activated in the entire organism.

### DXM Affects PGs Expression in Organotypic Hippocampal Culture *ex vivo*

**FIGURE 1:**
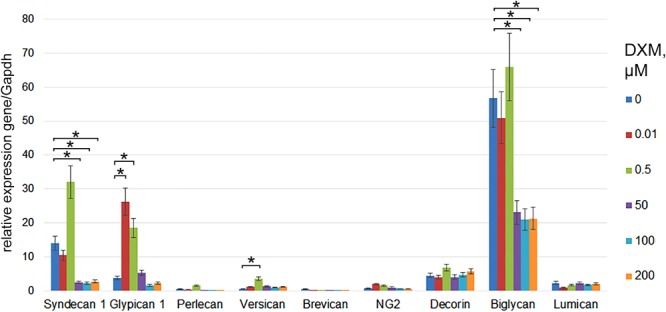
PGs expression levels in organotypic rat hippocampus cultures *ex vivo* before and after treatment with various concentrations of DXM. RT-PCR analysis, intensity of the amplified DNA fragments normalised to that of *Gapdh*. Bars represent the mean ± SD from triplicate experiments (OriginPro 8.1). Student’s *t*-test, ^∗^*p* < 0.05.

Rat brain sections were incubated with various concentrations of DXM (0.01–200 μM) for 24 h. The concentration lies between the minimal (0.001 μM) and maximal (500 μM) concentrations described in the literature (Kuwahara et al., 2003; Mulholland et al., 2005; Graber et al., 2012). Expression of main proteoglycan core proteins in the OHSC samples before and after DXM treatment was determined using RT-PCR (**Figure 1**). Low and high doses of DXM resulted in different changes in the expression of individual PGs compared with the control organotypic culture-low dose (0.01–0.5 μM) treatments increased expression levels of glypican-1 and versican core proteins (5-fold and 10-fold, respectively), whereas high dose (50–200 μM) treatments suppressed syndecan-1 and biglycan expression (5-fold and 3-fold, respectively). The results demonstrate a complex attenuation of PGs expression in normal rat brain by different DXM concentrations in the experimental model *ex vivo* and warrant further investigation of the effects *in vivo*.

### Proteoglycans Expression in Rat Brain Is Age- and Brain Zone-Dependent

**FIGURE 2:**
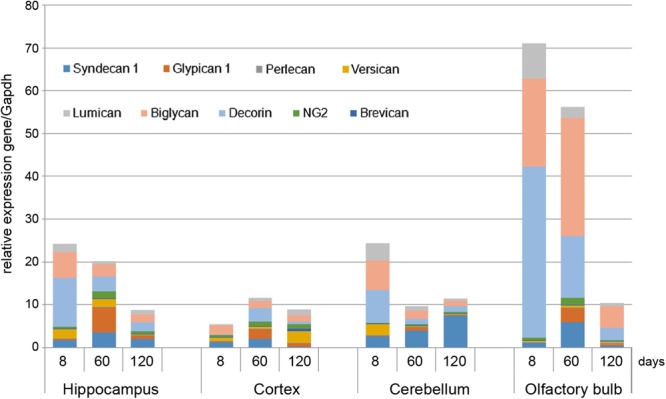
Proteoglycans expression in different brain zones of 8, 60, and 120-day old Wistar rats. RT-PCR analysis, intensity of the amplified DNA fragments normalised to that of *Gapdh* from triplicate experiments, Student’s *t*-test (OriginPro 8.1). Stacked column compares the contribution of each value to a total across categories.

As *in vivo* experiments are usually performed using 2-month old rats whereas OHSC study *ex vivo* was performed using hippocampi from 7 to 8 days old rat pups. Age-specificity as well as brain zone-specificity of the PGs expression levels were determined for a correct comparative analysis of the obtained data. Wistar rats aged 8, 60, and 120 days were used in the experiment and expression of main PG core proteins was profiled in various brain zones like hippocampus, cortex, cerebellum, and olfactory bulbs (**Figure 2**). According to the RT-PCR data, PGs demonstrated brain zone-specific expression patterns and overall transcriptional activities of the PGs core proteins in 8-day old rat pups. During post-natal development, significant changes were revealed for both parameters, consisting in continuous overall decrease of the PGs expression in the aged brains mainly due to decorin and biglycan down-regulation and tendency to syndecan-1 and glypican-1 up-regulation (especially at the age of 60 days).

The described effects were characteristic for most of the brain structures studied (hippocampus, cerebellum, and olfactory bulbs) except cortex, where moderate (1.5–2-fold) activation of the PGs expression was observed over 8–120 days development of the animals (**Figure 2**; **Table 2**). Taking into account a long period of drug administration, younger mice (60 days) look more suitable for the planned long-term experiments. So, 2-month old Wistar rats were used for further analysis of DXM and TMZ effects on PGs expression in hippocampus and cortex of normal rat brain *in vivo*.

**Table 2:** Expression of proteoglycans in various Wistar rat brain zones at different age.

	days	*Sdc1*	*Gpc1*	*Hspg2*	*Vcan*	*Bcan*	*Cspg4*	*Dcn*	*Bgn*	*Lum*
Hippocampus	**8**	**1.5** 0.2	0.33 0.05	0.08 0.00	2.0 0.2	NO	0.52 0.03	11.0 0.5	5.8 0.3	1.92 0.09
	**60**	3.4 0.1	5.9 0.2	0.12 0.00	1.9 0.7	0.06 0.00	1.71 0.06	3.4 0.1	2.93 0.7	0.72 0.01
	**120**	2.4 0.6	0.6 0.2	0.16 0.00	0.34 0.07	0.01 0.00	0.7 0.2	1.9 0.4	1.6 0.2	1.2 0.3
Cortex	**8**	1.17 0.06	0.2 0.02	0.05 0.00	0.91 0.04	NO	0.37 0.05	0.22 0.01	1.9 0.2	0.31 0.05
	**60**	1.9 0.7	2.24 0.08	0.2 0.1	0.47 0.03	0.21 0.05	1.0 0.1	3.16 0.01	1.69 0.03	0.73 0.06
	**120**	0.14 0.02	1.2 0.4	0.02 0.01	2.4 0.6	0.6 0.2	1.4 0.4	0.55 0.08	1.4 0.2	1.6 0.4
Cerebellum	**8**	2.5 0.1	0.22 0.00	0.05 0.00	2.8 0.1	NO	0.30 0.04	7.63 0.01	6.6 0.3	4.6 0.7
	**60**	3.9 0.6	0.9 0.1	0.05 0.01	0.15 0.02	0.08 0.01	0.30 0.06	1.2 0.1	2.0 0.2	1.1 0.1
	**120**	6.6 0.9	0.25 0.06	0.08 0.01	0.29 0.04	NO	0.3 0.1	1.2 0.2	1.30 0.01	0.7 0.1
Olfactory bulb	**8**	1.08 0.01	0.07 0.01	0.08 0.01	0.34 0.07	0.01 0.00	0.52 0.08	37.4 3.6	20.0 0.9	7.9 0.4
	**60**	5.9 0.7	3.4 0.1	0.08 0.02	0.6 0.03	0.01 0.00	1.66 0.01	14.5 0.5	27.7 0.9	2.46 0.05
	**120**	0.30 0.01	0.27 0.04	0.49 0.05	0.47 0.00	NO	0.23 0.03	3.3 0.6	5.03 0.01	0.9 0.2

*Expression calculated using 2^dCt^ method as gene/Gapdh ratio, NO, extremely low PG expression. Average expression levels (bold) ± SD are presented (OriginPro 8.1).*

### High-Dose DXM Treatment Significantly Affects Pattern and Transcriptional Activity of PG-Coding Genes in Rat Brain, Unlike Low DXM Doses or TMZ

**FIGURE 3:**
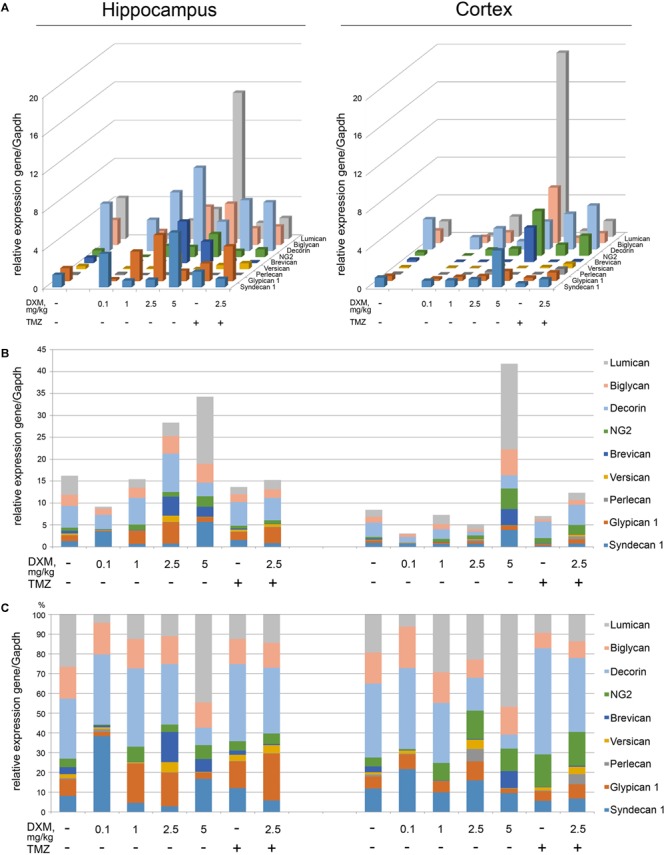
Proteoglycan mRNA expression levels in hippocampus and cortex of 2-month Wistar rats before and after treatments with DXM and/or TMZ. **(A)** mRNA expression levels of individual proteoglycan genes. **(B)** Overall transcriptional activity of the total pool of the studied PGs. **(C)** Expression pattern of the individual PG core protein-coding gene as percentage of 100%. RT-PCR analysis, intensity of the amplified DNA fragments normalised to that of *Gapdh*. Bars represent the mean ± SD from triplicate experiments, Student’s *t*-test (OriginPro 8.0).

The experimental animals were subjected to the treatment with different DXM concentrations (single injection with 0.1 or 2, 5 or 5 mg/kg DXM; repetitive treatment with 1 mg/kg DXM for a week, once/day), or TMZ treatment at the regimen close to that used for GBM patients, or both in combination. DXM concentrations range was taken from the literature data and adjusted in each part of the study (*ex vivo* and *in vivo*) because of the significant difference between the tissue level of DXM in human brain tumors and its’ cytotoxic concentration in cell culture (Nestler et al., 2002). After that, rat brains were dissected into the different morphological zones, and proteoglycan expression was detected in hippocampus and cortex by RT-PCR analysis (**Figure 3**). Syndecan-1, glypican-1, decorin, biglycan, and lumican were identified as the most expressed proteoglycan-coding genes both in normal rat hippocampus and cortex (**Figure 3A**). High-dose DXM treatments (2.5 and 5 mg/kg) resulted in the increased overall transcriptional activity (**Figure 3B**) and specific expression patterns (**Figure 3C**) of the PGs in hippocampus in dose-dependent manner, mainly due to changes in syndecan-1 (+4-fold), glypican-1 (+3-fold), brevican (+7-fold), CSPG4/NG2 (+2-fold), decorin (-2-fold), and lumican (+3-fold) expression.

Interestingly, the PGs expression in cortex was more resistant to DXM treatment, and only highest DXM concentration (5 mg/kg) significantly increased the transcriptional activity of the genes (**Figure 3B**). In common, long-term treatment by low doses of DXM (1 mg/kg) looks more favourable in terms of PGs expression in normal brain tissue for both hippocampus and cortex than high DXM doses.

TMZ almost did not affect overall transcriptional activities of the PG core protein-coding genes in both brain zones-neither alone nor in combination with the selected DXM concentration (2.5 mg/kg) (**Figure 3B**), although that modified PG expression patterns both in hippocampus (increase of glypican-1 and decrease of lumican expression levels) and cortex (increase of HSPG2/perlecan and CSPG4/NG2 expression levels) (**Figure 3C**).

Totally, high doses of DXM (5 and 2.5 mg/kg in a less extent) demonstrated pronounced effects towards the activation of transcriptional activity of PG-coding genes and changes in their expression pattern in rat brain tissue, while moderate DXM dose (1 mg/kg) did not affect significantly the mRNA levels of the studied PGs. TMZ alone or in combination with 2.5 mg/kg DXM treatment also did not result in significant changes in the expression of PG core proteins in normal rat brain (both hippocampus and cortex) at the transcriptional level.

### Suppression of the Overall Proteoglycans Expression in Normal Brain Tissue Varies for Different TMZ-Based Drugs

**FIGURE 4:**
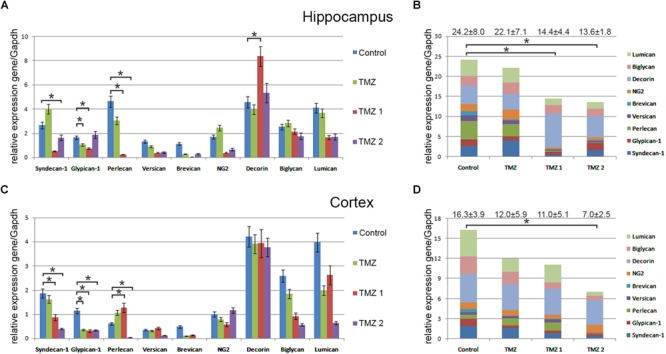
Proteoglycan expression levels in hippocampus and cortex of 2-month Wistar rats before and after treatments with different TMZ-based drugs. **(A,B)** Hippocampus, **(C,D)** Cortex. **(A,C)** mRNA expression levels of individual proteoglycan genes. RT-PCR analysis, intensity of the amplified DNA fragments normalised to that of *Gapdh*, bars represent the mean ± SD from triplicate experiments, Student’s *t*-test (OriginPro 8.1). **(B,D)** Overall transcriptional activity of PG core proteins in different TMZ-treated groups of animals. Stacked column compares the contribution of each value to a total across categories. Proteoglycan mRNA expression levels in hippocampus and cortex of 2-month Wistar rats before and after treatments with DXM and/or TMZ. mRNA expression levels of individual proteoglycan genes in hippocampus **(A)** and cortex **(C)**. Overall transcriptional activity of the total pool of all studied PGs in hippocampus **(B)** and cortex **(D)**. Expression pattern of the individual PG core protein-coding gene as percentage of 100%. RT-PCR analysis, intensity of the amplified DNA fragments normalised to that of *Gapdh*. Bars represent the mean ± SD from triplicate experiments (OriginPro 8.0). ^∗^*p* < 0.05.

Interestingly, various TMZ-based drugs demonstrated different effects on the overall transcriptional activity and expression pattern of proteoglycan-related genes (**Figure 4**). Common trend was a down-regulation of the overall proteoglycan expression in both hippocampus and cortex, up to 2-fold depending on the drug (TMZ, TMZ1, or TMZ2). The most affected PG type were HSPGs (syndecan-1, glypican-1, and perlecan), being the main contributors in the overall inhibition of the PGs expression by some of the drugs (TMZ1 and TMZ2). The demonstrated effects of the TMZ-based drugs were more pronounced for hippocampus than the cortex of normal rat brain.

### Low Dose of DXM and TMZ Affect Proteoglycan Content in Brain ECM at Core Protein Level

**FIGURE 5:**
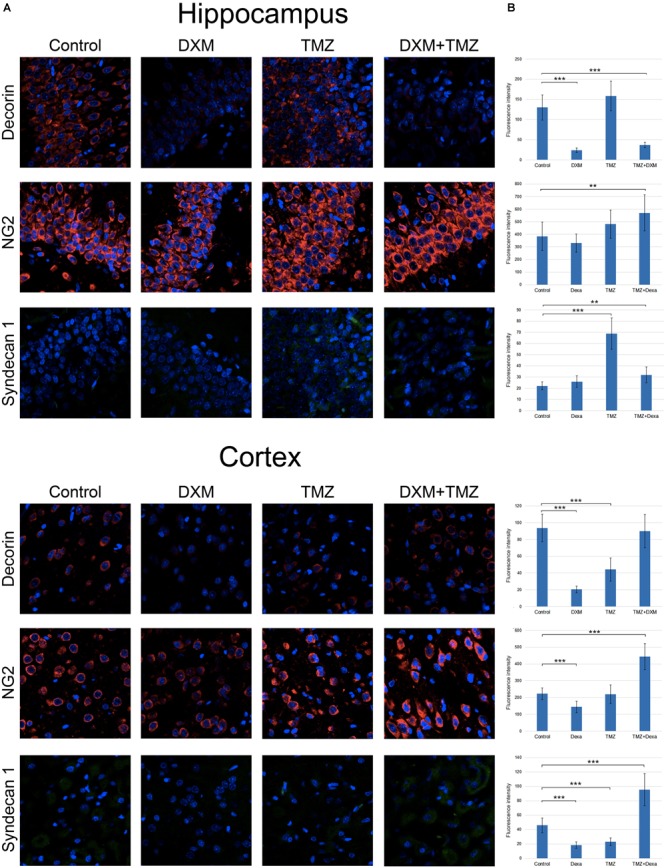
Immunofluorescence analysis of PG core proteins content in rat brain before and after treatments with DXM and/or TMZ. **(A)** Immunostaining with anti-decorin, anti-CSPG4/NG2, or anti-syndecan-1 antibodies. Visualization with secondary antibodies Alexa Fluor 647 (red, for decorin and CSPG4/NG2) or Alexa Fluor 488 (green, for syndecan-1). Magnification ^∗^400. **(B)**. Quantitative analysis of the PG core proteins content in the control and treated rat brain tissues (CellProfiler 2.2.0 software). Student’s *t*-test, ^∗^*p* < 0.05, ^∗∗^*p* < 0.01 and ^∗∗∗^*p* < 0.001.

To investigate a potential effect of the treatments onto the PGs expression at the protein level, immunofluorescence analysis of the selected PG core proteins (syndecan-1, decorin and CSPG4/NG2) was performed for control rat brains and those treated with DXM (2.5 mg/kg on days 1 and 4 of the experiment), TMZ (30 mg/kg per day for 5 days) or both (**Figure 5**; **Table 3**). These PGs were chosen from the PGs with the highest expression levels in the normal rat brain tissue (both in hippocampus and cortex), and as representatives from main proteoglycan subtypes (heparan sulphate proteoglycan, chondroitin sulphate proteoglycan and dermatan sulphate proteoglycan). The obtained results revealed differential effects of the drugs onto PG core proteins content in different brain zones. DXM treatment resulted in the decrease of decorin staining both in hippocampus and cortex (5-fold, *p* < 0,001 and 4,5-fold, *p* < 0,001, respectively), whereas TMZ significantly increased expression of syndecan-1 (3-fold, p < 0,001) in hippocampus but not cortex. There was a tendency for increased expression of CSPG4/NG2 in hippocampus upon the TMZ treatment as well; however, the change was not statistically significant (**Figure 5**; **Table 3**).

**Table 3:** Immunofluorescence analysis of individual PG core proteins and GAG content in hippocampi and cortex of control and DXM and/or TMZ-treated Wistar rats.

		Decorin	Syndecan-1	Cspg4/NG2	HS	CS
Hippocampus	Control	130.2 8.9	22.1 1.9	385.4 35.8	22.7 2.5	36.4 3.2
	DXM	24.0 1.9	25.9 1.8	330.9 24.7	63.2 5.8	63.3 4.5
	TMZ	158.5 12.4	68.9 5.4	481.0 36.1	53.1 6.3	65.0 5.2
	TMZ + DXM	36.9 3.1	32.0 2.3	570.4 44.9	34.4 2.7	86.5 6.2
Cortex	Control	93.6 11.4	45.8 4.7	222.4 15.2	99.0 13.5	41.7 6.2
	DXM	20.49 1.7	18.3 1.8	144.3 12.6	58.3 6.1	20.6 2.0
	TMZ	44.2 7.4	22.9 2.4	220.3 21.4	41.1 5.3	46.9 5.7
	TMZ + DXM	89.9 10.5	95.3 10.9	444.6 33.6	250.9 34.1	202.7 25.1

*Average fluorescence intensity calculated using Cell Profiler 2.2.0 software. HS, heparan sulphate and CS, chondroitin sulphate.*

Interesting, the most profound effects were detected for the combined treatment (DXM + TMZ), with brain zone-specific characteristics. In hippocampus, significant decrease of decorin core protein content (3-4-fold, *p* < 0,001) along with an evident increase of CSPG4/NG2 (0,5-fold, *p* < 0,01) were observed. In cortex, up-regulation of CSPG4/NG2 and syndecan-1 (2-fold, *p* < 0,001 and 2-fold, *p* < 0,001, respectively) and no changes in decorin content were detected.

The results demonstrate an overall deterioration of the proteoglycan composition in the DXM and/or TMZ treated normal brain tissues at core protein level mainly due to changes in decorin, CSPG4/NG2 and syndecan-1 content. The changes in the proteoglycan core proteins upon DXM/TMZ treatment(s) stay in line with the RT-PCR data on more profound effects of the studied drugs towards hippocampus rather than cortex, and represent side effects of the conventional anti-glioblastoma therapy towards the normal rat brain tissue in the experimental system *in vivo*.

### Both DXM and TMZ Significantly Increase Glycosaminoglycan Content in Rat Brain, Especially in Combination

**FIGURE 6:**
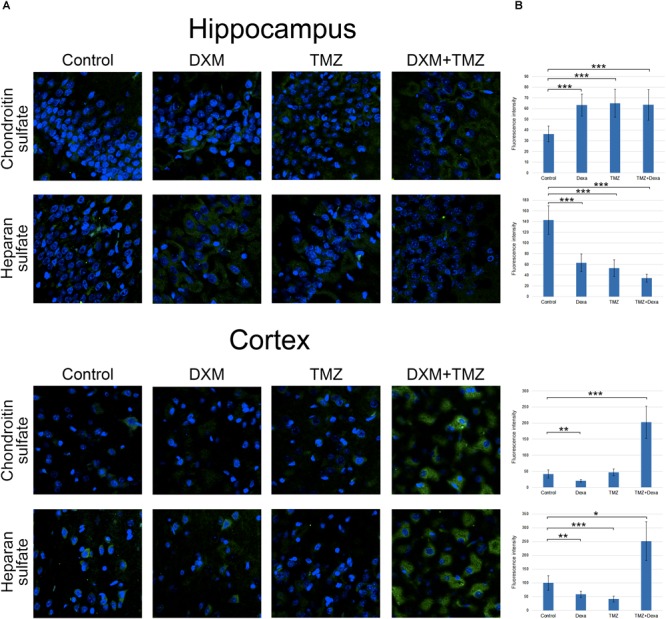
Immunofluorescence analysis of GAGs content in rat brain before and after treatments with DXM and/or TMZ. **(A)** Immunofluorescence analysis with anti-HS and anti-chondroitin sulphate antibodies. Visualization with secondary antibodies Alexa Fluor 488 (green). Magnification ^∗^400. **(B)** Quantitative analysis of the GAGs content in the control and treated rat brain tissues (CellProfiler 2.2.0 software). Student’s *t*-test, ^∗^*p* < 0.05, ^∗∗^*p* < 0.01 and ^∗∗∗^*p* < 0.001.

Along with core proteins, polysaccharide chains of GAGs [heparan sulphate (HS) and chondroitin sulphate (CS)] represent an important structural and functional part of the entire proteoglycan molecules. To determine potential effects of DXM and TMZ onto polysaccharide content of normal brain tissue, specific antibodies to the polysaccharide epitopes on HS and CS molecules were used for immunofluorescence analysis of the control and DXM and/or TMZ treated rat brain tissues (**Figure 6**).

Separate treatment with DXM or TMZ resulted in increase of HS and CS content in hippocampus (2-3-fold and 2-fold, respectively, *p* < 0,001) but not cortex. However, the most pronounced effects were observed for the combined DXM/TMZ treatments, which resulted in the significant increase of HS and CS content in hippocampus (1,5-fold, *p* < 0,01 and 2-2,5-fold, *p* < 0,001, respectively) and especially cortex (2,5-fold and 5-fold, respectively, *p* < 0,001).

Taken together, the obtained results for the first time demonstrate an ability of DXM and TMZ to affect PG/GAG expression and composition in normal rat brain tissue in the experimental system *in vivo*. The revealed changes might contribute to transformation of normal brain ECM into tumorigenic niche, susceptible for the enhanced invasion of the residual glioma cells and tumour progression.

## Discussion

The presented results demonstrate that anti-glioblastoma drugs TMZ and DXM affect the content and expression pattern of PGs in normal rat brain tissue *ex vivo* and *in vivo*. As to TMZ, in spite of the known anticancer effects of the drug, there are no published data on its’ possible action onto the normal cells or tissues to compare with. A different situation is about DXM, which is often used as anti-oedema drug during anticancer therapy. Its toxic side effects are demonstrated for many different cancers including glioma (Shields et al., 2015; Murayi and Chittiboina, 2016), where DXM may decrease the effectiveness of treatment and shorten survival of GBM patients, substantiating the request for restricted use of corticosteroids in glioblastoma (Pitter et al., 2016). However, molecular mechanisms of the DXM side effects remain unclear.

In the literature, we did not find any direct data about DXM or TMZ effects onto PGs content/composition in normal brain tissue to compare with, unlike their effect towards glioblastoma cells or GBM tumours. It is known that the expression levels of many PGs are changed significantly in glioblastoma cells and during the tumour development. For example, multiple changes occur in gliomas with a common tendency to overall activation of the expression of various PG core proteins: syndecan-1 and perlecan is significantly up-regulated in malignant glioma cell lines and GBM specimens (Watanabe et al., 2006); high expression of glypican-1 in glioma endothelial cells is associated with cell cycle progression (Qiao et al., 2008) and high expression of perlecan is associated with poor GBM prognosis (Kazanskaya et al., 2018); brevican is highly up-regulated in gliomas and promote cancer cells motility in primary brain tumours (Hu et al., 2008); CSPG4/NG2 is expressed in gliomas in oncofoetal manner and is involved in disease progression (Stallcup and Huang, 2008); significant (3–15-fold) up-regulation of CD44 and NG2 expression during the experimental glioma development in mouse glioma model *in vivo* suggest the molecules as possible molecular markers of tumour invasion (Wiranowska et al., 2006), however, human GBM specimens study supports NG2 but not CD44 as a possible prognostic markers of glioblastoma progression (Tsidulko et al., 2017); CSPGs can serve as critical regulators of glioma cells invasion and play an important role in organisation of tumour microenvironment (Silver et al., 2013).

The demonstrated ability of the conventional anti-glioblastoma chemotherapy to modulate PG and GAG expression and/or composition in normal brain tissue, for the first time reveals these brain ECM molecules as potential microenvironmental biomarkers for DXM side effects or molecular targets to search for perspective anti-DXM neuroprotective drugs. From the practical point of view, it underlines a necessity for the possible revision of the current DXM use during the GBM treatment. Moderate dose (1 mg/kg) seems to be the most appropriate in terms of consistency of PGs expression in the brain of the control and treated animals and possibly should be taken as a desirable dose. It means long-term treatment with low dose of DXM could be more appropriate that single high-dose DXM injection to preserve the transcriptional activity of PG-coding genes and resulted structure of brain ECM. The results are indirectly supported by the demonstrated DXM effects in organotypic culture of animal normal brain *ex vivo*: DXM exposure after oxygen-glucose deprivation potentiates oxygen-glucose deprivation-mediated cytotoxicity in organotypic cerebellar slice cultures prepared from neonatal rat pups (Mulholland et al., 2005); DXM is able to suppress transcription of AVP gene during stimulation by cAMP (Kuwahara et al., 2003).

Another interesting finding is that the high or lower doses of DXM have different “targets” on the complex proteoglycan molecules–whether the first one affects transcriptional activity of the PG core proteins, the last one points to polysaccharide GAG chains of the molecule. This fact makes the revealed GAG changes worse detected in the routine research or diagnostic studies, hiding this aspect of the side effects of the widely used drugs like DXM. Important aspect could be related as well to a potential contribution of the DXM-induced changes in normal brain tissue (and especially its ECM) into the neurological, psychological, and social problems for long-term survivors of glioblastoma (Gately et al., 2017). The demonstrated increase of the GAGs content in TMZ/DXM-treated normal brain tissue can be related to an unfavourable transformation of brain ECM into tumourigenic niche supporting glioma cells growth. The data stay in line with the available data on this issue: HS content is significantly increased in the relapsed GBM tumours (Kazanskaya et al., 2018); disruption of cell–cell and cell–ECM interactions by chondroitinase ABC treatment enhances the chemotherapeutic availability and sensitivity of glioma cells to TMZ (Jaime-Ramirez et al., 2017); glioblastoma- and neuroblastome-derived CSC-like cells with high expression of decorin and lumican are resistant to TMZ suggesting a novel pivotal role for the PGs in drug resistance and cell plasticity of glioma stem cells (Farace et al., 2015); blocking of GAG function during anticancer therapy could be a perspective strategy to improve cancer treatment (Belting, 2014).

**FIGURE 7:**
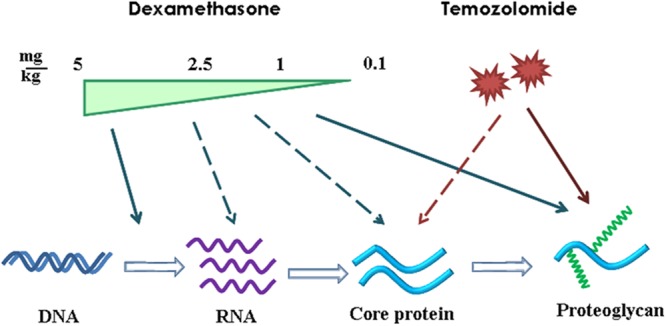
Schematic representation of the effects of DXM and TMZ onto PGs expression and content in normal rat brain tissue in the experimental system *in vivo*. Arrows–significant effect and dotted arrows–weak effect.

Comparative analysis of the results, obtained at the transcriptional level of PGs expression (RT-PCR), core proteins expression (IHC) and HS/CS polysaccharide content (IHC with anti-HS/CS antibodies to the polysaccharide epitopes) suggests complex multi-level molecular mechanism of DXM/TMZ effects onto PGs content and distribution in brain tissue in rat experimental model *in vivo* (**Figure 7**). The effects are clearly dose-dependent, high DXM doses result in deregulation of the transcriptional activity of PG-coding genes, whereas low DXM doses and TMZ affect predominantly polysaccharide GAG chains of the molecules.

In summary, the demonstrated disorganization of proteoglycan expression and content in normal rat brain tissue under the DXM/TMZ pressure might contribute to formation of favourable microenvironment for proliferation and invasion of the remaining post-surgery glioblastoma cells, resulting in relapse of the disease. The obtained results extend our knowledge in this field and for the first time reveals the DZM/TMZ-induced changes in PGs expression and ECM organization in normal brain tissue as one of the possible molecular mechanisms of known toxic side effects of the conventional anti-glioblastoma therapy.

## Author Contributions

AT and TP carried out organotypic culture experiments *ex vivo*. AT and AS performed the animal experiments. AT and GdLB performed RT-PCR analyses. CB, AS, GK, and SA performed immunostaining. EG and AT designed the experiments and wrote the manuscript.

## Conflict of Interest Statement

The authors declare that the research was conducted in the absence of any commercial or financial relationships that could be construed as a potential conflict of interest.
